# Genetic Characterization of the *Drosophila* Birt-Hogg-Dubé Syndrome Gene

**DOI:** 10.1371/journal.pone.0065869

**Published:** 2013-06-17

**Authors:** Wei Liu, Zhi Chen, Yansen Ma, Xiaochun Wu, Yaping Jin, Steven Hou

**Affiliations:** 1 Key Laboratory of Animal Biotechnology, the Ministry of Agriculture, College of Veterinary Medicine, Northwest A&F University, Yangling, Shaanxi, China; 2 The Mouse Cancer Genetics Program, National Cancer Institute at Frederick, National Institutes of Health, Frederick, Maryland, United States of America; Cardiff University, United Kingdom

## Abstract

Folliculin (FLCN) is a conserved tumor suppressor gene whose loss is associated with the human Birt-Hogg-Dubé (BHD) syndrome. However, its molecular functions remain largely unknown. In this work, we generated a *Drosophila* BHD model through genomic deletion of the FLCN gene (*DBHD^−^*). The *DBHD* mutant larvae grew slowly and stopped development before pupation, displaying various characteristics of malnutrition. We found the growth delay was sensitive to the nutrient supplies. It became more severe upon restrictions of the dietary yeast; while high levels of yeast significantly restored the normal growth, but not viability. We further demonstrated that leucine was able to substitute for yeast to provide similar rescues. Moreover, the human FLCN could partially rescue the *DBHD^−^* phenotypes, indicating the two genes are involved in certain common mechanisms. Our work provides a new animal model of the BHD syndrome and suggests that modulation of the local nutrient condition might be a potential treatment of the BHD lesions.

## Introduction

The BHD syndrome is a rare genetic disorder that is clinically characterized by frequent lung cysts, benign hair follicle tumors, and a high risk to develop kidney cancers. Inactivation of the folliculin (FLCN) gene is the genetic basis of BHD syndrome. FLCN is present in a wide range of organisms, from the single-cell yeast to human, indicating it may regulate certain basic cellular processes. However, its biological functions are still not clear [1]–[3].

Several FLCN mutant cell lines and animal models have been developed to unravel its functions. One discovery from these works is the intriguing relations between FLCN and the mechanistic target of rapamycin (mTOR), a highly conserved nutrient sensor among eukaryotes whose mutations have been found in certain human diseases including cancer [4]. The first clue of interactions between FLCN and mTOR came from biochemical works. Through purification of a FLCN interacting protein (FNIP), people found FLCN was a potential target of the 5′AMP-activated protein kinase (AMPK) and mTOR [5]. This was later realized to be a consequence of possible feedback mechanism, as the same group observed overactivated mTOR in the hyperplastic kidneys from the FLCN knock-out mice (FLCN*^−^*) [6], [7]. Surprisingly, other researchers observed mTOR was both up- and down-regulated in certain FLCN mutant cell lines and the FLCN*^−^* mouse tissues [8], [9]. At present, how FLCN interacts with mTOR is still not determined. Another solid observation is that the FLCN*^−^* mice stopped development at very early embryonic stages with severely disorganized structures [6]–[9]. The cause of the failed embryogenesis is not known yet and it has not been linked with other abnormalities in adults. Recently, people characterized an anti-apoptosis function of FLCN through the TGF-β pathway, which was proposed to be a new mechanism to account for its tumor suppressor roles [10], [11].

*Drosophila* provides an ideal model system to study the genotype-phenotype relationships. Its genome contains a single FLCN homologue gene (*DBHD*). Using an RNAi-mediated gene knockdown assay, people uncovered a role of *DBHD* in the male germline stem cell maintenance and suggested the dysregulated stem cell homeostasis might be a potential mechanism of the BHD tumorigenesis [12]. The RNAi is a method to partly suppress gene functions, while most genetic lesions of the reported BHD cases are FLCN loss [3]. Therefore, it is better to use null mutant to model the pathological conditions. For this reason, we generated a *DBHD* knockout allele (*DBHD^−^*). The *DBHD* mutant larvae displayed various features of malnutrition, including growth retardation, small body size and larval lethality. The growth defects, but not the lethality, could be significantly rescued by dietary yeast or the branched-chain amino acid of leucine. We further demonstrated that the rescue effect is likely a consequence of elevated dTOR signaling, because a specific dTOR signaling suppressor, rapamycin, could reverse the rescues of *DBHD^−^* mutants by yeast or leucine. Moreover, the human FLCN could partially rescue the *DBHD^−^* mutants, indicating at least some molecular functions of the two homologous genes are conserved. Our work provides a novel animal model of the BHD syndrome and suggests that modulation of the local nutrient conditions deserves further investigations for treatment of BHD.

## Materials and Methods

### Generating the DBHD Knockout Fly

To make the *DBHD* targeting construct, two genomic fragments from both sides of the *DBHD* locus, about 5 kb each, were amplified and inserted into the *NotI* and *AscI* cloning sites of the pW25 vector [13]. PCR amplification primers: TCTTTTTAGGCGCGCCTACACTTGGGCCTCC and TGACCAGGCGCGCCCATTCACTGAAATACCAG (with *AscI* site); CAATCCGCGGCCGCTTTCACTGATAAAAACGAG and GACTATGCGGCCGCAATCATTGATGAGGGGTTG (with *NotI* site). The screening procedure was performed according the protocol described before [13].

### Making the DBHD Rescue Constructs a DBHD Polyclonal Antibody

To make the *DBHD-res*, a genomic fragment covering the complete *DBHD* locus till the adjacent gene (CG14829) was amplified from the *Drosophila* genomic DNA. PCR amplification primers: GCACTCTAGACCACAGGTAATGAACAG and GCTGTCTAGACTGGATTCGGCATC. EGFP tag was then fused in frame with either terminus of the *DBHD* transcription unit by fusion-PCR. The human FLCN cDNA (a kind gift of Laura S. Schmidt) was amplified by PCR and cloned into the pUAST vector to make the UAS-hFLCN transgene. To generate a polyclonal DBHD antibody, the EcoRI-XhoI fragment of DBHD cDNA, which encodes the N-terminal 88 aa from N2 to L89, was amplified by PCR and inserted into the expression vector pGEX4T1. This construct was transfected into bacteria BL21 for expressing GST-fusion protein. The purified GST-fusion protein was injected into rabbit to generate antiserum against DBHD. On SDS-PAGE, it recognizes a major band at about 55 kDa, the same size as the predicted DBHD protein.

### Fly Stocks and Food Preparation

In the *DBHD* mutant screening experiment, we used the following stocks: *y w; 70FLP, 70I-SceI* (BL#6934) and *w^1118^; 70FLP* (BL#6938). The following flies were used to generate mosaic clones: *hsp-flp; RRT80B ubi-GFP/FRT80B DBHD^−^* and *eyeless-flp; FRT80B ubi-GFP/FRT80B DBHD^−^*. We crossed the following flies to rescue *DBHD^−^* with hFLCN: *hsp-Gal4, DBHD^−/^TM3, Sb* and *UAS-hFLCN; DBHD^−/^TM3, Sb*. The normal food recipe used in our lab: 8% sugar, 10% corn flour, 1.5% baker’s yeast, 1% agar, 0.4% Propionic Acid and 0.1% Nipagin. Chemicals used in the feeding experiments: 3-methyl adenine (Invitrogen); Rapamycin (LC Laboratories). Leucine, arginine, glutamine, tryptophan, cholesterol, and riboflavin were all bought from Sigma. For the free amino acids analysis, the third instar larvae were rinsed with 70% ethanol and allowed to be air-dried. More than 55 larvae were homogenized followed by sonication. Proteins were precipitated by sulfosalicyclic acid. After centrifugation, the supernatant were passed through 0.22 µm filter and analyzed using the Hitachi automatic amino acid analyzer L-8900.

### Immunocytochemistry

The following primary antibodies were used: rabbit anti GFP (1∶500, Invitrogen); rabbit anti PH3 (1∶1000, Invitrogen). Mouse anti Armadillo (1∶200) and Prospero (1∶200) were from DSHB. Guinea pig anti-Dpn (1∶1000) was a gift from Jim Skeath. ApopTag-Red assay kit was from Millipore. The fluorescent secondary antibodies, LysoTracker, and Click-iT EdU assay kit were all bought from Invitrogen.

## Results

### Gene Targeting of DBHD

**Figure 1:**
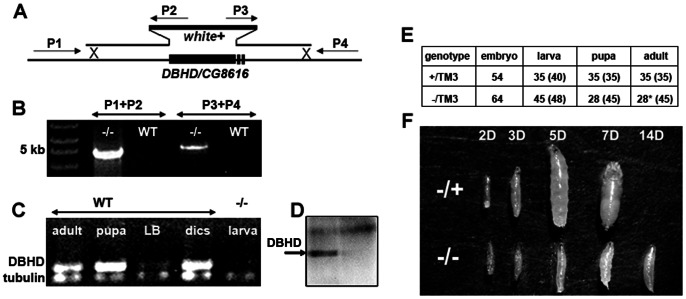
Generating a *DBHD* null allele. (A) The *DBHD* genomic locus and the targeting strategy. P1–P4 represent the PCR primers. (B) PCR analysis of the genomic DNA. In *DBHD^−/−^* larvae (−/−), a 4.8 kb fragment and a 5 kb fragment could be amplified by the corresponding primer pairs. (C) rtPCR analysis of the *DBHD* transcripts in various tissues. LB: larval brain; disc: mixtures of larval imaginal discs. α-tubulin (at 84B) was used as the positive control. (D) A DBHD antibody recognizes a band at about 55 kDa (arrow) of the whole larval extracts, which is absent in the *DBHD^−/−^* larvae. *w^1118^* was used as the wild-type control (WT). (E) Statistical analysis of the developmental profiles of two strains. The +/TM3 flies contains a healthy 3rd chromosome and a GFP-marked third chromosome balancer (*TM3, Kr::GFP*). The −/TM3 flies contains the same balancer and the *DBHD^−^* allele. Animals survived to different stages were counted. Numbers in the parenthesis are the theoretical values according to the Mendel rules. *: all the survived adults are heterozygotes. (F) Comparison of *DBHD^−/−^* (−/−) and the sibling heterozygotes (−/+) at different days after egg laying. Embryos collected within three hours and cultured in the same food vials were picked for images at each time point. All heterozygotes have eclosed by 14 days after egg laying and thus were not pictured.

To genetically ablate the *DBHD* function, we used the homologous recombination strategy to delete the *DBHD* genomic sequence within the germ cells from living animals (“ends-out”, 13). The *DBHD* gene encodes a 460-amino-acid protein, spanning 1712 base pairs (bp) on the left arm of chromosome 3 with three exons. The targeting construct contains a 4.8 kb and a 5 kb of genomic fragments flanking the *DBHD* transcription unit (**Figure 1A**). It was firstly introduced into the fly genome through the standard P-element-mediated transformation. The targeting cassette was later released and linearized from the germ cell genome by two endogenously produced enzymes (Flipase and I-SceI, 13). Following homologous recombination, the complete exon1 of *DBHD* including sequences encoding the first 400 amino acids and the 5′ untranslated region (5′ UTR) will be replaced with a *white* marker gene. This should give rise to a *DBHD* null allele (**Figure 1A**). Because the BHD gene is conserved in a wide range of organisms and the BHD knockout mice die at very early embryonic stages, we suspected that *DBHD* was a vital gene. To this end, we screened about 500 gametes and uncovered a lethal allele on chromosome-3 where *DBHD* resides.

We performed several experiments to check the mutation. We first did PCR analysis and confirmed the targeting effects on both arms as predicted (**Figure 1A, B**). RT-PCR results further revealed the *DBHD* transcript was present at various developmental stages, but was absent in the homozygous mutants (**Figure 1C**). We also generated a *DBHD* polyclonal antibody. In the western blot experiment, it recognized a major band at about 55 kDa of the total larval extracts, which was missing in the mutant samples (arrow in **Figure 1D**). Finally, we made transgenic flies harboring an exogenous genomic fragment containing the complete *DBHD* exons and the upstream non-transcribed sequences till the adjacent gene (referred to as *DBHD-res*, see the later section for the structures). One copy of *DBHD-res* could rescue the mutants to healthy adults without any obvious abnormalities compared with the heterozygotes. These results revealed that we obtained a clean null allele of *DBHD*.

### DBHD is an Essential Gene

The homozygous *DBHD* mutants (hereafter referred to as *DBHD^−/−^*) never survived to adults. To trace the developmental progresses, we combined the *DBHD^−^* mutant allele with a GFP-marked balancer chromosome (referred to as −/*TM3, Kr::GFP*). Firstly, we wanted to check if the mutation had any dominant effects. We compared the developmental profiles of the heterozygotes (−/*TM3, Kr::GFP*) and another strain containing an isogenized wild-type third chromosome (recovered from single *w^1118^* fly) with the same balancer chromosome (*+/TM3, Kr::GFP*). Embryos were collected within three hours and allowed to develop in stable environment (25°C, 60% humidity). Under such conditions, the heterozygotes behaved similarly with the wild-type controls (including both +/+ and +/TM3, *Kr::GFP*) by developing into healthy adults at around the same time point (**Figure 1E**). Thus, *DBHD* is essentially a recessive gene.

Based on the statistical analysis and direct check under fluorescence microscope, we found that the *DBHD^−/−^* embryos (negative of *Kr::GFP*) hatched normally (**Figure 1E, F**). However, when most heterozygous larvae have entered into the third instar stage three days later, all *DBHD^−/−^* larvae were much smaller than their sibling heterozygotes (**Figure 1F**). The mean weight of a heterozygous larva at third instar is about 0.0013 g (n = 273). For *DBHD^−/−^*, this number is about 0.0003 g (n = 185). The *DBHD^−/−^* larvae could survive for a prolonged period of time (up to three weeks). Eventually, all mutants died as small larvae. We separately cultured the mutants and the heterozygotes in different food vials. Each vial contained no more than ten newly hatched larvae. This should provide each individual with sufficient food and space, and minimize the potential toxicities brought by the crowds or heterozygotes. The *DBHD^−/−^* larvae still showed the same growth retardation phenotype, which could rule out the weakness in intraspecific competition as a causal mechanism.

Some *Drosophila* zygotic mutants can survive the embryogenesis because of the large amounts of maternal gene products deposited in the eggs. To exclude the potential maternal contributions, we generated *DBHD^−/−^* germline clones by removing the *DBHD* products at the beginning of egg formation [14]. Animals developed from the *DBHD^−/−^* germline clones showed no difference with the zygotic mutants in development: they successively passed through the embryo stage and died before pupation. We conclude that *DBHD* plays essential roles in the larval stages.

### Mitosis and Endoreplication were Suppressed in *DBHD^−/−^* Larvae

After hatching from eggs, the *Drosophila* larvae feed continuously to increase their body mass dramatically before the onset of pupation. The larval imaginal and endoreplicative cells make the main contributions to this change. The imaginal cells, including those in the imaginal discs, the gonads and the brain, go through active mitosis to increase the cell number. In contrast, the endoreplicative cells from the gut, salivary gland and fat body, increase the DNA polyploidy and cell volume without further cell divisions [15]. We dissected the 4-day-old larvae and checked both the imaginal and the endoreplicative tissues.

**Figure 2:**
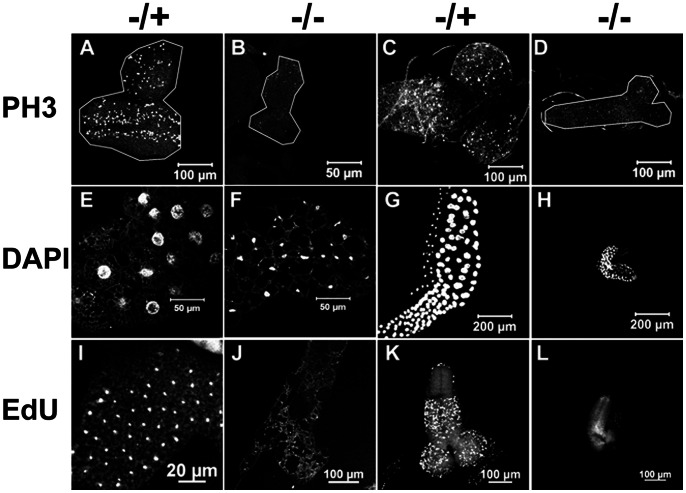
Mitosis and endoreplication are suppressed in *DBHD^−/−^* larvae. PH3 marks the mitotic cells. EdU marks the cells undergoing DNA synthesis. DAPI marks the nuclei. (A and B): Eye imaginal discs. (C, D, K, L): Brains. (E, F, I, J): Fat bodies. (G and H): Salivary glands. The sibling heterozygotes (−/+) were taken as the wild-type controls. Note all the *DBHD^−/−^* samples (−/−) are reduced in size, the polyploidy are also reduced in cells from fat body (F) and salivary gland (H).

Most *DBHD^−/−^* larvae had tiny or even no visible imaginal discs. The brains were also reduced in size (**Figure 2**). These phenotypes could be caused by decreased cell division, increased cell death or both. We analyzed the apoptosis in larval brains (ApopTag, Invitrogen). No clear differences were observed between the larval brains of mutants and heterozygotes (unpublished observation). The Phospho-Histone H3 (PH3) is a reliable mitotic cell marker by labeling the condensed chromosomes during mitosis. We found the PH3-positive cells were dramatically declined in various imaginal tissues of the *DBHD^−/−^* larvae (**Figures 2A–D**). Thus, cell division is suppressed in the *DBHD* mutants.

Another obvious discrepancy was that the mutant larvae were less opaque than their heterozygote siblings. The latter were normally filled with white fat body, a nutrient storage and sensing organ that is equivalent to the mammalian liver/adipose tissues. We found that the *DBHD^−/−^* larvae had very thin fat bodies. In addition, the mutant cells were filled with large vacuoles and their nuclei seemed to be shrunken, which were in contrast with the large polyploid nuclei in heterozygotes (**Figure 2E, F**). Similarly, both the cell volumes and DNA contents of the salivary gland cells were also markedly reduced (**Figure 2G, H**). We used the EdU incorporation assay, a thymidine analogue, to label the DNA synthesis. In the early third instar heterozygotes, a large number of the imaginal cells and endoreplicative cells were undergoing DNA replication. In contrast, the reaction was rather quiescent in the *DBHD^−/−^* tissues (**Figures 2I–L**). Taken together, a combined suppression of cell division and cell growth should account for the small body phenotype of the *DBHD^−/−^* larvae.

### Autophagy is Elevated in the *DBHD^−/−^* Larvae

**Figure 3:**
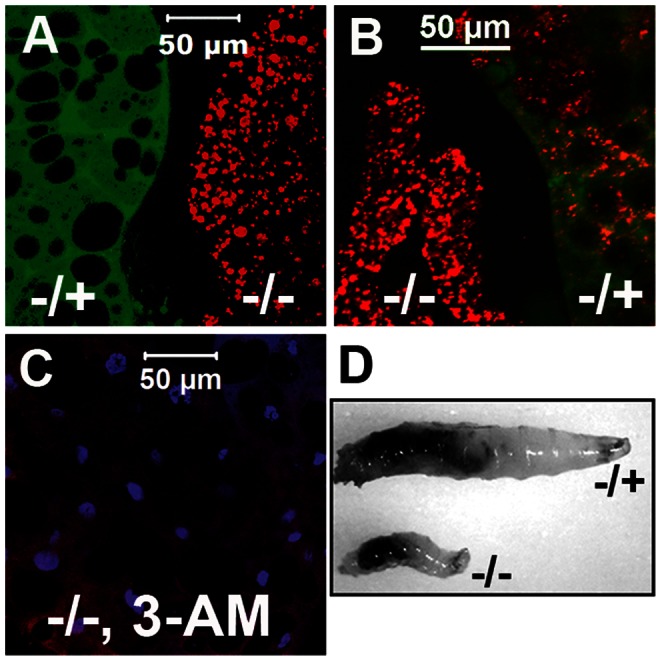
Autophagy is elevated in the *DBHD^−/−^* larvae. (A–C) LysoTracker staining (red) of unfixed larval fat bodies. The GFP-positive tissues (green) are from heterozygotes (*Kr::GFP*). (A) The LysoTracker signal is much stronger in the *DBHD^−/−^* (−/−) fat bodies than in the heterozygotes (−/+). The 4-day-old larvae were picked form the same food vial, processed in the same staining tube and imagined in one optical field. (B) LysoTracker signal became strong in the heterozygotes starved for 3 hours by supplying with distilled water only. The same staining of fat body from starved *DBHD^−/−^* is also shown. (C) 3-Methyladenine (3-MA) suppresses autophagy in *DBHD^−/−^* fat body. (D) Foraging assay by feeding larvae with colored food (the baker’s yeast powder mixed with black ink). Green: GFP (A–C); Blue: DAPI (C).

The above *DBHD^−/−^* phenotypes were reminiscent of starvations or mutations that blocked the nutrient-sensing signaling pathways in *Drosophila*. It is known that upon nutrient restrictions, especially the dietary protein, autophagy is induced in the fat bodies by degradation of the non-essential cell organelles to supply critical nutrients for survival [15]. We stained the freshly dissected fat body with a red-fluorescent dye to mark autophagy (LysoTracker, Invitrogen). Under normal feeding conditions, the LysoTracker signal is very faint in the heterozygotes. In contrast, it is greatly elevated in the *DBHD^−/−^* larvae (**Figure 3A**). The heterozygous larvae showed stimulated autophagy upon nutrient starvation for several hours (**Figure 3B**). To check if the strong autophagy in *DBHD^−/−^* larvae is responsible for their growth defects, we fed them with 3-Methyladenine, an autophagy inhibitor [16]. It efficiently suppressed autophagy (**Figure 3C**), while it could not restore the normal growth or viability in the mutants. Thus, elevated autophagy alone is not sufficient to cause the growth defects in *DBHD^−/−^* larvae.

To check if the above starvation-like phenotypes are caused by foraging difficulties, we fed larvae with colored yeast paste. The *DBHD^−^* mutants behaved similarly with controls in swallow and excretion. Both of them were attracted by this nutritious food and successively passed it into the midgut (**Figure 3D**). Once they were put back to the clean food, the colors within the guts were quickly cleaned out. Thus, the *DBHD^−/−^* larvae do not have obvious defects to obtain food and excrete the waste. All the above experiments were repeated with the *DBHD^−/−^* germline clones to remove maternal influences and similar results were obtained.

### The Growth Delay of *DBHD^−/−^* Larvae was Significantly Rescued by Yeast-rich Food

**Figure 4:**
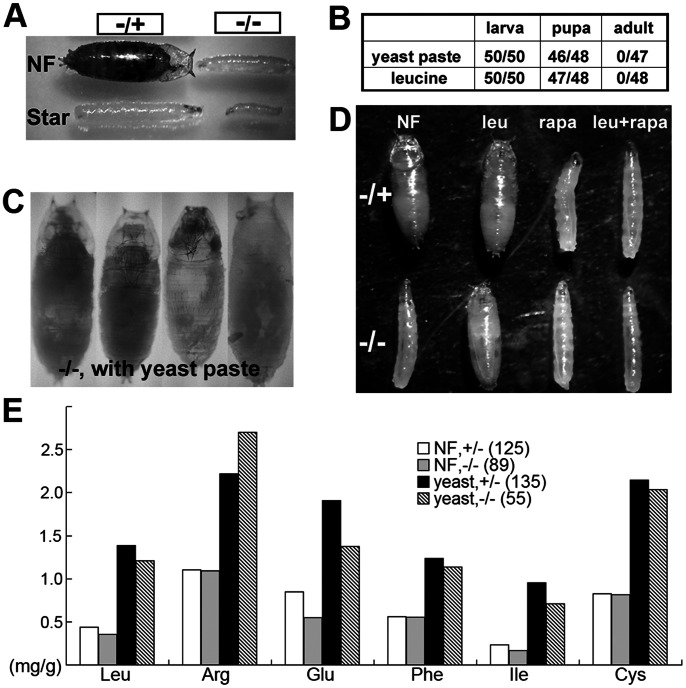
Rescue of the *DBHD^−/−^* growth defects by nutrients. (A) *DBHD^−/−^* larvae were sensitive to yeast supply. NF: normal food; Star: starvation, normal food without yeast. All samples were picked at Day 8 after hatching from eggs. (B) The developmental profiles of flies cultured on two kinds of nutritious foods. yeast paste: the baker’s yeast powder was mixed with water and supplied on agar plate. leucine: normal food supplemented with 100 mM leucine. Fifty newly hatched larvae for each genotype were picked. Numbers of the heterozygotes (left) and *DBHD^−/−^* were separated by slashes. (C) Examples of four dead *DBHD^−/−^* pharates cultured on yeast pastes. (D) Rescue effects of leucine. Animals aged for 7 days after hatching were imaged. NF: normal food; leu: normal food with 100 mM leucine; rapa: normal food with 1 µM rapamycin; leu+rapa: normal food with 100 mM leucine and 1 µM rapamycin. (D) Free amino acids analysis of the larvae. The amino acids levels are displayed as milligram per gram of body weight (mg/g). The amounts of larvae for each experiment are listed in the parenthesis.

Because the *DBHD^−/−^* larvae show certain starvation-like phenotypes, we sought to investigate their growth responses to different nutrient conditions. Dietary yeast is the major source of nutrients in fly food. Firstly, we cultured the newly hatched larvae on less nutrient food as described before (normal food recipe without yeast, [14]). The heterozygotes grew slowly on this kind of medium (**Figure 4A**). Interestingly, the mutants became even smaller than the mutants fed with normal food (**Figure 4A**), suggesting the *DBHD^−/−^* animals are sensitive to the yeast supplies.

Next, we checked their growth responses to nutrient food. We picked no more than twenty newly hatch larvae, put them on culture medium with different concentrations of yeast and analyzed their developmental profiles (**Table 1**). As expected, the heterozygotes took longer time for eclosion with diluted food or yeast-free food. Once the yeast concentration was above it of the normal food recipe, the heterozygotes showed similar growth rate. Surprisingly, the yeast-rich food significantly reversed the growth retardation phenotypes of the *DBHD^−/−^* larvae. The yeast-only food brought the best rescues, no matter it was living or dead (autoclaved).

**Table 1:** The developmental profiles of flies cultured on various yeast foods.

	−/+	−/−
normal food (1.5% yeast)	eclosed, 8–9D	no pupation
diluted food (0.75% yeast)	eclosed, >14D	no pupation
yeast-free food	eclosed, >15D	no pupation
rich food-1 (10% yeast)	eclosed, 8–9D	pupated, 5–6D
rich food-2 (20% yeast)	eclosed, 8–9D	pupated, 5–6D
yeast paste	eclosed, 8–9D	pupated, 4–5D
yeast paste +5% sugar	eclosed, 8–9D	pupated, 4–5D
yeast paste on normal food	eclosed, 8–9D	pupated, 5–6D

Note: The developmental profiles were displayed by listing the last stages that they survived and the time point when they started to enter (days after hatching). Pure yeast paste had the best rescue effects. Diluted food means the food nutrients (sugar, corn flour and baker’s yeast) were 50% of the normal food recipe. Yeast-free food has normal food recipe without yeast. In all test, we picked the newly hatched larvae at the same time point. No more than twenty larvae were cultured within each food chamber. At least 100 larvae in total were counted for each experiment.

On pure yeast paste, nearly all mutants could grow into fat third instar larvae and successively pupate at more or less the same time point as heterozygotes (**Table 1** and **Figure 4B**). Some mutant phenotypes, including the increased autophagy, suppressed mitosis and endoreplication of larval cells, were also mostly rescued. However no mutant eclosed, they mostly died at the pupal stage. Some dead pupae even developed discernable adult structures including bristles, legs, wings and eyes, suggesting *DBHD* had no gross influences on cell fate specifications (**Figure 4C**). Therefore, nutrient is an effective factor to rescue the growth defects in *DBHD^−/−^* larvae.

### Leucine is Able to Substitute for Yeast to Restore the Normal Growth of *DBHD^−/−^* Larvae

Yeast is the major source of three groups of nutrients in fly food, including cholesterol, vitamins (especially the B family) and amino acids. The larval growth will be delayed if any of these components is limited [17]–[20]. To find out the active gradient(s) in yeast responsible for the rescues of *DBHD^−/−^* larvae, we cultured flies on normal food supplemented with single nutrient (**Table 2**). For all the components that we tested, only leucine provided comparable rescues with the yeast-rich food. With additional leucine, most *DBHD^−/−^* larvae successfully pupated and died during metamorphosis (**Table 2** and **Figure 4D**). The rescue effects were consistent at all the concentrations we tested (10, 50, 100 and 500 mM). Other essential amino acids, including tryptophan, arginine or glutamine, did not show obvious rescues at high concentrations (100 mM). High levels of tryptophan were even toxic by suppressing the larval growth further (**Table 2**, [21]). We conclude that the leucine-mediated mechanism is responsible for the rescue of *DBHD* ^−/−^ larvae.

**Table 2:** Rescue of the *DBHD^−/−^* larvae by supplemented nutrients.

Culture media	developmental profile
Normal food	no pupation
+Riboflavin (0.1 mg/ml)	no pupation
+cholesterol (40 mM)	no pupation
+Leucine	most died as pupae
+Tryptophan	no pupation
+Arginine (100 mM)	no pupation
+Glutamine (100 mM)	no pupation

Note: All tested components were supplemented as additions in the normal food. Leucine and tryptophan were tested at various concentrations (10, 50, 100, and 500 mM). Supplementation of leucine provided consistent rescues at all tested concentrations. Excessive tryptophan further inhibited growth at high concentrations (>100 mM). At least 200 embryos collected within three hours were tested.

### The Gross Absorption of Amino Acids is not Impaired in *DBHD^−/−^* Larvae

The foraging assay revealed that the *DBHD^−/−^* larvae fed normally. Next, we checked their abilities of food digestion and amino acid absorption. The third instar larvae were homogenized. The concentrations of 16 free amino acids from the whole extracts (including those in the haemolymph and cytoplasm) were measured using the automatic amino acid analyzer (Hitachi, L-8900). With either normal food or yeast paste, no significant differences were observed between the heterozygotes and the *DBHD^−/−^* larvae (6 amino acids are selectively shown in **Figure 4E**). This result further demonstrates that food digestion and the gross absorption of amino acids are not apparently impaired in the *DBHD^−/−^* larvae.

### Rapamycin Suppressed the Rescue Effects of Leucine on *DBHD^−/−^* Larvae

Amino acids are important stimulators of mTOR signaling, among which leucine is the most efficient [22], [23]. We noticed that the *DBHD^−/−^* phenotypes, including small body size, growth delay and larval lethality, were indeed similar to those of the *dTOR* mutants [24], [25]. Therefore, we checked if *dTOR* signaling is responsible for the rescue of *DBHD^−/−^* larvae.

Rapamycin is a specific inhibitor of the mTOR signaling. Flies fed with a low dose of rapamycin displayed the starvation-like phenotypes [24]. We used the same concentration of rapamycin in the food (1 µM) and found that it efficiently suppressed the rescue effects of leucine or yeast paste (**Figure 4D** and data not shown), all the *DBHD^−/−^* larvae eventually died before pupation. We propose that active *dTOR* signaling is responsible for the rescue of growth defects in *DBHD^−/−^* larvae.

### The Human FLCN could Perform Partial DBHD Functions

**Figure 5:**
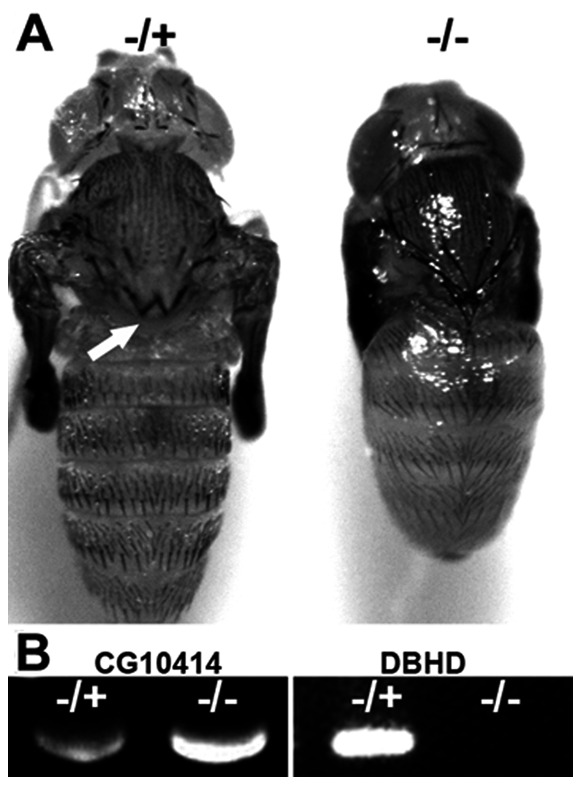
The human FLCN could partially rescue the *DBHD^−/−^* larvae. (A) Dorsal view of pupae. The heterozygote (−/+) is revealed by *Sb* (marked with short and thick bristles on the notum, arrow), see materials and methods for the cross scheme. (B) The genotypes of the pupae were confirmed by PCR analysis of genomic DNA. The fly CG10414 gene was used as a positive control.

To investigate if the roles of *DBHD* are conserved in mammals, we attempted to rescue the *DBHD^−/−^* flies with human FLCN (hFLCN). We generated a UAS-hFLCN transgenic fly and expressed the human FLCN gene under the control of *hsp-Gal4* driver. The newly hatched larvae fed with normal food were heated for 30 minutes at 37°C, twice a day, to ubiquitously induce the expression of hFLCN. No rescued *DBHD^−/−^* flies eclosed. However, some *DBHD^−/−^* larvae expressing *hsp/hFLCN* (about one third) could develop into pupae with no clear defects of the cuticles (**Figure 5**). Because 46% of the amino acids of DBHD and hFLCN proteins are similar [12], and we used a ubiquitous expression driver, it is not so surprising that we obtained only partial rescues. Nevertheless, this result reveals that the hFLCN could at least perform partial *DBHD* functions, suggesting the two genes are involved in common mechanisms.

### DBHD is Expressed Broadly

**Figure 6:**
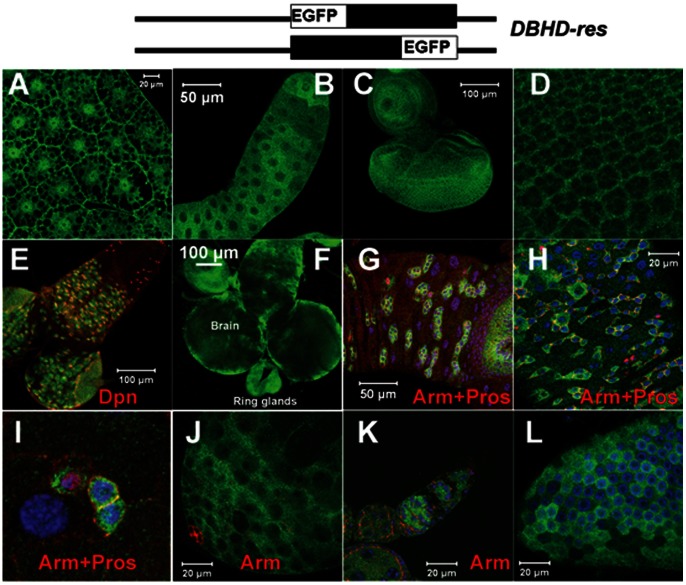
Expression patterns of *DBHD-res*. The expression of *DBHD-res* was detected by the EGFP staining (green). Genotype in all panels: *DBHD-res*; *DBHD^−/−^*. (A) Larval fat body. (B) Larval salivary gland. (C) eye-antennal disc. (D) Magnified view of eye disc. (E) Larval brain. (F) Ring glands. (G) Epithelium of larval midgut. (H) Epithelium of adult midgut. (I) Magnified view of adult midgut. (J) Testis. (K) Germarium. (L) Egg follicles. Arm (red) marks the cell borders; Prospero (Pros, red, in nuclei) marks the intestinal EE cells; Dpn marks the neuroblasts in the brains; DAPI (blue) marks the nuclei in G–I, L. Note the *DBHD-res* was also expressed in some EC cells in the gut (E–G, marked by the DAPI-labeled polyploid cells).

We made two *DBHD* rescue constructs in which the EGFP tag was fused in frame with either terminus of the *DBHD* transcription unit (**Figure 6**, together referred to as *DBHD-res*). The transcriptions were under the control of native *DBHD* promoter and the upstream sequence till the adjacent gene. Either transgene could fully rescue the *DBHD^−/−^* animals into healthy adults. Therefore, the EGFP signal should be able to monitor the essential localizations of DBHD proteins. Both transgenes showed the same EGFP expression patterns, suggesting the DBHD proteins functioned in full length throughout their lifetime.

We used the rescued homozygous mutants (*DBHD-res*; *DBHD^−/−^*) to check the expression of *DBHD-res*, so that there were no endogenous DBHD proteins. The EGFP is broadly present in larval phases, including the imaginal discs, fat bodies, central nervous system (CNS) and midguts (**Figure 6**). It is expressed in fat bodies, in both nuclei and cytoplasm (**Figure 6A**). It is also concentrated in the cytoplasm of the entire eye, wing and leg imaginal disc cells. Using a neuroblast marker of *Deadpan* (*dpn*) [26], we detected *DBHD-res* in most, if not all neuroblasts in the larval CNS (**Figure 6E**). It has specific patterns in the midguts throughout larval and adult stages (**Figures 6G–I**), where it is mainly in the cytoplasm of diploid cells, including intestinal stem cells (ISCs), enteroblast cells (EBs) and enteroendocrine cells (EEs). It is also apparently expressed in many, but not all polyploid enterocytes (ECs). In adults, it is also enriched in the tips of both testis and germarium, and the nutritive follicle cells of the eggs (**Figures 6J–L**).

### DBHD is Not Required Cell-autonomously for the Growth of Larval Imaginal Disc Cells

**Figure 7:**
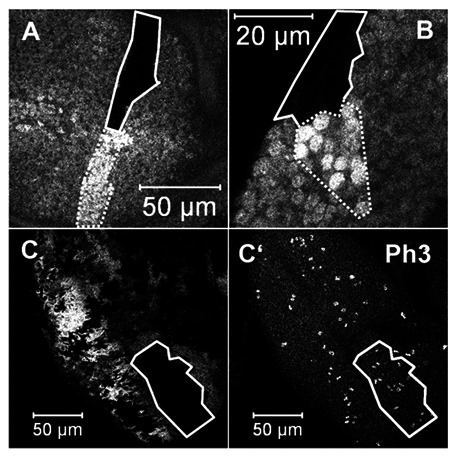
Clonal analyses of *DBHD^−/−^* cells in the larval imaginal discs. (A–C) GFP signals. (C’) PH3. Random clones were generated in the wing (a) and eye (b) imaginal discs. *eyeless-flipase* induced large clones in the eye disc (C, C’). The *DBHD^−/−^* cells are absent of GFP (green) and circled with solid lines. The wild-type twin spot cells are marked with double GFP signals and enclosed with dashed lines. Note the *DBHD^−/−^* and the twin spots are similar in clone size. The amount of PH3-positive cells is not clearly declined or increased in the *DBHD^−^* clones.

The *Drosophila* larval imaginal discs are active proliferative tissues, which will develop into the adult appendages during morphogenesis. They provide excellent systems to study the mechanisms of cell proliferation and cell fate specifications. We generated *DBHD^−/−^* clones in the larval imaginal discs. Surprisingly, the mutant clones were similar in size to the wild-type twin spots (**Figures 7A, B**). The cell numbers in the twin clones did not show clear differences (counted by DAPI signals). Therefore, the *DBHD^−/−^* cells do not have growth advantages over their sibling wild-type cells. In addition, we did not find any morphological defects associated with the mutant cells in adults. We also used the *eyeless-flipase* to generate large *DBHD^−/−^* clones in eye discs, again there were not obvious phenotypes in adult eyes and mitosis seemed normal within the mutant clones (**Figures 7C, C** **’**). These results suggest that *DBHD* is not required cell-autonomously in these tissues.

## Discussion

In summary, we developed a new animal model of the BHD syndrome and demonstrated that some functions of FLCN are conserved in *Drosophila* and mammals. An interesting discovery from our fly model is that the growth defects of *DBHD* mutant larvae could be substantially rescued by nutrient. It is, therefore, of great interest to investigate if modulation of the local nutrient conditions is beneficial for the treatment of BHD lesions in mammalian systems.

It is not clear why nutrient (particularly leucine) could rescue the growth defects of *DBHD^−/−^* animals. *DBHD* either functions in parallel with, or is directly involved in the leucine-mediated mechanisms. Because the normal food can support the growth of heterzygotes, but not *DBHD^−/−^*, we speculate that leucine must play roles other than protein synthesis. So far, the best known role of leucine as a signaling factor is to activate mTOR [21], [22]. In consistent with this, inhibition of *dTOR* by rapamycin reversed the rescue effects of leucine or yeast.

If *DBHD* is involved in *dTOR* signaling, as it does in mammals, we propose here one mechanism that *DBHD* functions to sequester amino acids within cellular organelles to activate *dTOR* in some *Drosophila* tissues. *DBHD* does not encode a typical membrane protein and *DBHD-res* is mainly expressed in the cytoplasm of several cell types (**Figure 6**). The *DBHD^−/−^* larvae could feed, and obtain sufficient nutrient from the yeast-rich foods, implying the food digestion and gross amino acids absorption are not severely impaired. Recently, it was found that mTOR needs to be translocated to the surfaces of lysosomes for activation [27]. It is thus possible that *DBHD* helps to accumulate leucine within the lysosomes to evoke dTOR. This locally enriched leucine could be alternatively achieved by saturation mechanism through increasing its supply in the food. Further experiments are definitely required to clarify the mechanism.

Unlike mice, *DBHD* is not required for the embryonic development. This is nevertheless consistent with a role of *DBHD* in sequestering amino acids from the environment. The *Drosophila* embryos rely on the nutrients deposited in the eggs entirely. It is not until the larval stage that they start to obtain nutrients from the food. The eggs might contain sufficient leucine to support the embryogenesis of *DBHD^−/−^*, which takes about only 24 hours. After hatching, the *DBHD^−/−^* larvae fed with nutritious food could pupate. However, as the leucine is gradually consumed during metamorphosis (about 96 hours), they eventually stop development before eclosion.

It is surprising that *DBHD* controls the growth of the imaginal disc cells in a non-cell-autonomous manner, which does not support a classical anti-tumor function. Our work suggests that at least in the *Drosophila* imaginal discs, *DBHD* controls growth through some neuronal/hormonal mechanisms. Consistently, *DBHD-res* is expressed in various endocrine cells or tissues, including the gut epithelia, fat body, brain, and ring glands (**Figure 6**). The latter is an endocrine organ to secrete hormones including juvenile hormone (JH) and the *Drosophila* insulin-like peptides (DILPs). Flies missing these products will develop the starvation-like phenotypes. At present, we are analyzing the functions of *DBHD* in these places.
